# Employment Loss and Food Insecurity — Race and Sex Disparities in the Context of COVID-19

**DOI:** 10.5888/pcd19.220024

**Published:** 2022-08-18

**Authors:** Jacquelyn V. Coats, Sarah Humble, Kimberly J. Johnson, Havisha Pedamallu, Bettina F. Drake, Elvin Geng, Charles W. Goss, Kia L. Davis

**Affiliations:** 1 Brown School at Washington University in St. Louis, St. Louis, Missouri; 2 Brown School at Washington University, School of Medicine, Department of Surgery, St. Louis, Missouri; 3 Brown School at Washington University, School of Medicine, Division of Infectious Diseases, St. Louis, Missouri; 4 Brown School at Washington University, School of Medicine, Division of Biostatistics, St. Louis, Missouri

## Abstract

**Introduction:**

Applying an intersectional framework, we examined sex and racial inequality in COVID-19–related employment loss (ie, job furlough, layoff, and reduced pay) and food insecurity (ie, quality and quantity of food eaten, food worry, and receipt of free meals or groceries) among residents in Saint Louis County, Missouri.

**Methods:**

We used cross-sectional data from adults aged 18 or older (N = 2,146), surveyed by using landlines or cellular phones between August 12, 2020, and October 27, 2020. We calculated survey-weighted prevalence of employment loss and food insecurity for each group (Black female, Black male, White female, White male). Odds ratios for each group were estimated by using survey-weighted binary and multinomial logistic regression models.

**Results:**

Black female residents had higher odds of being laid off, as compared with White male residents (OR = 2.61, 95% CI, 1.24–5.46). Both Black female residents (OR = 4.13, 95% CI, 2.29–7.45) and Black male residents (OR = 2.41, 95% CI, 1.15–5.07) were more likely to receive free groceries, compared with White male residents. Black female (OR = 4.25, 95% CI, 2.28–7.94) and White female residents (OR = 1.93, 95% CI, 1.04–3.60) had higher odds of sometimes worrying about food compared with White male residents. Black women also had higher odds of always or nearly always worrying about food, compared with White men (OR = 2.99, 95% CI, 1.52–5.87).

**Conclusion:**

Black women faced the highest odds of employment loss and food insecurity, highlighting the disproportionate impact of COVID-19 among people with intersectional disadvantages of being both Black and female. Interventions to reduce employment loss and food insecurity can help reduce the disproportionately negative social effects among Black women.

**Summary**

**What is already known on this topic?**

COVID-19 has widened existing sex and racial disparities that affect the health of adults in the US. Studies have shown food insecurity and employment loss are not evenly distributed across sociodemographic groups.

**What is added by this report?**

Few studies have examined how race, ethnicity, and sex intersect to affect employment loss and food insecurity in a metropolitan location of the US.

**What are the implications for public health practice?**

Results can be used to guide programs, interventions, and policy to mitigate the disproportionate effects of COVID-19 and related social harms on Black women.

## Introduction

Employment and food insecurity have been identified as 2 critical social determinants of health and health equity (1). Women and people of color have historically been at greater risk for both (2,3). Since the beginning of the COVID-19 pandemic, these long-standing social, economic, and health inequities that disproportionately affect women and people of color have intensified (4,5). However, the depth and breadth of the pandemic’s effects on already socioeconomically marginalized groups need assessment.

A well-established body of literature documents the link between employment loss and adverse health outcomes, including increased risk of death, substance abuse, psychological distress, suicide, and unmet health care needs (6–8). People facing employment loss may simultaneously be at greater risk for food insecurity because of economic hardship. Additionally, food insecurity has been associated with poor diet quality and decreased access to healthy food options, such as fruits and vegetables (9); unfavorable mental health outcomes, including elevated stress, depression, and anxiety (10–11); substandard physical health status (11); and chronic disease (12).

Early evidence also indicates adverse mental and physical health consequences resulting from employment loss and food insecurity since the COVID-19 pandemic began (6). Although evidence on the effects of COVID-19 on food insecurity and employment is mounting, few studies have examined the potential harms of the pandemic by using an intersectional approach. Analyzing the effects of COVID-19 using an intersectionality framework can highlight how multiple social identities (eg, race, gender, class) might interact to influence health outcomes among segments of the population that would otherwise remain hidden (13,14). We aimed to fill this gap and by investigating the effects of COVID-19 on sex and racial inequality in employment and food security outcomes. We used data to analyze the social needs and harms associated with COVID-19 on employment and food insecurity for adults by race and sex in Saint Louis County, Missouri. This study is part of larger research that estimated the prevalence of COVID-19 infections in the region with a secondary aim to assess how the pandemic affected their lives across a variety of domains. Additional details on the parent research have been published elsewhere (15). 

St. Louis County has almost 1 million residents, with 52.6% of residents identifying as female, 60.3% as female, 60.3% aged 18 to 64 years, and 17.6% aged 65 years or older, respectively (16). Non-Hispanic White residents make up 66.0% of the county’s total population while non-Hispanic Black residents account for 24.1% (16). Most adult residents have a high school diploma (49.9%) or a higher level of education (43.7%) (16). The median household income is $67,420, with incomes for White households above the median at $77,989 and incomes for Black households below the median at $43,801 (16). During our study period, it was estimated that approximately 7.5% of all county residents had been infected with the COVID-19 virus, with infection rates among Black residents nearly 3 times higher than White residents (15). This disparity is comparable with nationwide trends that report higher COVID-19 cases and deaths among Black people.

## Methods

### Eligibility and recruitment

We used a combination of random digit dialing (RDD) and targeted-telephone sampling from Marketing Systems Group (https://www.m-s-g.com/Pages/), a commercial vendor to recruit 2,314 participants from August 12, 2020, and October 27, 2020. Eligible participants included residents of St. Louis County, Missouri, aged 18 years or older who were available by landline or cellular telephone. We oversampled telephone numbers tied to county locations with a majority of Black residents in an attempt to obtain equal Black and White resident participation. Participation in the study involved testing for COVID-19 infection or participation in an approximate 15-minute telephone survey. This study was approved by the institutional review board of Washington University in St. Louis.

We conducted a sensitivity analysis to evaluate the impact of readjusting weights to reflect the reduced sample size compared with the sample from which the weights were originally derived. This analyses revealed that reweighting the data did not significantly change our statistical inferences or conclusions; therefore, we retained the original weights in our analysis.

### Measures

The telephone-administered survey assessed 11 topics including, demographics, testing willingness, health status and access, current chronic health conditions, tobacco use, and COVID-19–specific items. When appropriate, the survey included previously validated and tested items from the Behavioral Risk Factor Surveillance System (17).

### Sociodemographics

We collected self-reported sociodemographic information. Sex was categorized as female or male. Age was measured continuously in years. Race was categorized as Black, White, or other. Other racial and ethnic groups included American Indian/Alaska Native residents, Asian American/Native Hawaiian/Other Pacific Islander, and Hispanic residents. Because of their small sample size (n = 68), other racial groups were excluded from this analysis. Education status was categorized as high school diploma equivalent or less, some college (1–3 years), and college graduate (≥ 4 years). The number of children 18 years or younger living in participant households was dichotomized as no children and 1 or more children. Participants reported their annual household income from all sources (<$10,000, $10,000–$14,999, 15,000–19,999, $20,000–$24,999, $25,000–$34,999, $35,000–$49,000, $50,000–$74,999, ≥$75,000). Marital status was married, divorced, widowed, separated, never married, or member of an unmarried couple, and current employment status was employed for wages, self-employed, retired, or unemployed (including those out of work for less than 1 year, out of work for 1 year or more, homemaker, student, or unable to work). Health care coverage was determined by the participant as having any kind of health care coverage (including health insurance, prepaid plans, or government-sponsored plans) or none.

### COVID-19-related employment loss

Participants were asked a series of 3 yes or no questions on how their employment status was affected by the COVID-19 pandemic. We asked if they had been furloughed, laid off, or had their pay or hours reduced because of COVID-19.

### COVID-19–related food insecurity

The survey included 3 questions related to food insecurity since the beginning of the COVID-19 pandemic. We inquired about the quantity and quality of food eaten since the pandemic’s start by asking, “Which of these statements best describes the food eaten in your household since the COVID-19 pandemic started?” Response options were enough food, enough food but not type wanted, sometimes not enough food, or often not enough food. To assess the magnitude of worry about food, respondents were asked, “Since the beginning of the pandemic, have you worried that your food would run out before you buy more?” Response options included always, nearly always, sometimes, seldom, and never. Seldom or never worried were collapsed into one response. Finally, participants provided a yes or no response to the question, “Since the pandemic, did you or anyone in your household get free groceries or a free meal?”

### Statistical analysis

Survey respondents were assigned weights to be representative of the underlying population of St. Louis County with respect to sex, location, and sample type (RDD or targeted telephone sample). Before the weighting process, missing data for key variables were imputed by using hot-deck imputation. This technique handles missing data by replacing each missing value with an observed response from a comparable respondent. We first weighted the sample obtained through RDD by using a standard process and then combined the data with the targeted sample to be weighted to select variables in the survey. At each step, results were examined for extreme values and trimmed.

We calculated the survey’s weighted prevalence for each of the employment and food security outcomes for each race by sex population segment (Black female, Black male, White female, White male). Differences (*P* < .05) between groups were determined using the Rao-Scott χ^2^ test. We then conducted survey-weighted logistic regression models to calculate odds ratios and 95% CIs associated with the race-by-sex subgroups and each of our employment and food insecurity outcomes. Key sociodemographic variables associated with respondents included the presence of children in the home, age, education, and employment. Weighted multinomial logistic regression was used to calculate the odds ratios for associations with the quality of food and food worry outcomes. All analyses were performed by using SAS software version 9.4 (SAS Institute). R software version 4.1.2 (R Foundation for Statistical Computing) was used to create visuals.

## Results

### Descriptive statistics

A total of 2,246 respondents participated in the survey (Table 1). Among the sample, 1,421 respondents (63.3%) were female, 861 (38.3%) were Black, and 1,017 (45.3%) were aged 65 years or older. Black residents were less likely, compared with their White counterparts, to be college graduates (31.9% vs 61.2%) or be currently married (33.9% vs 58.7%). Approximately 28.6% of the overall sample had an income of <$35,000, with a higher proportion of Black respondents (44.5%) living below this threshold compared with White respondents (18.6%).

**Table 1:** Sample Demographics, by Race, in Surveyed Adults (N = 2,246) Living In St. Louis County, Missouri, August 12, 2020–October 27, 2020

Demographics	Overall, n (%)	White, n (%)	Black, n (%)
**Total**	2,246	1,385 (61.7)	861 (38.3)
**Sex**			
Female	1,421 (63.3)	822 (59.4)	599 (69.6)
Male	825 (36.7)	563 (40.6)	262 (30.4)
**Age (mean, SD)**	59.63 (16.6)	60.6 (16.6)	58.14 (16.5)
**Marital status**			
Married	1,105 (49.2)	813 (58.7)	292 (33.9)
Divorced	328 (14.6)	167 (12.1)	161 (18.7)
Widowed or separated	335 (14.9)	180 (13.0)	155 (18.0)
Never married or Other	478 (21.3)	225 (16.2)	253 (29.4)
**Education**			
High school diploma or less	500 (22.3)	216 (15.6)	284 (33.0)
College, no degree	624 (27.8)	322 (23.2)	302 (35.1)
College, undergraduate or advanced degree	1,122 (50.0)	847 (61.2)	275 (31.9)
**Employment status**			
Employed for wages	853 (38.0)	532 (38.4)	321 (37.3)
Self-employed	133 (5.9)	99 (7.2)	34 (4.0)
Out of work ≥1 years	48 (2.1)	22 (1.6)	26 (3.0)
Out of work <1 year	86 (3.8)	49 (3.5)	37 (4.3)
Persons working in household	47 (2.1)	35 (2.5)	12 (1.4)
Student	37 (1.7)	24 (1.7)	13 (1.5)
Retired	925 (41.2)	575 (41.5)	350 (40.7)
Unable to work	117 (5.2)	49 (3.5)	68 (7.9)
**Health care coverage**			
No	143 (6.4)	54 (3.9)	89 (10.3)
Yes	2,103 (93.6)	1,331 (96.1)	772 (89.7)
**Presence of children in the household**			
No	1,731 (77.1)	1,083 (78.2)	648 (75.3)
Yes	515 (22.9)	302 (21.8)	213 (24.7)
**Income, $**			
<10,000	73 (3.3)	21 (1.5)	52 (6.0)
10,000–$14,999	60 (2.7)	22 (1.6)	38 (4.4)
15,000–$19,999	117 (5.2)	35 (2.5)	82 (9.5)
20,000–$24,999	194 (8.6)	82 (5.9)	112 (13.0)
25,000–$34,999	198 (8.8)	98 (7.1)	100 (11.6)
35,000–$49,999	376 (16.7)	204 (14.7)	172 (20.0)
50,000–$74,999	405 (18.0)	254 (18.3)	151 (17.5)
>75,000	823 (36.6)	669 (48.3)	154 (17.9)

### Prevalence of employment loss and food insecurity by race and sex

**Figure 1:**
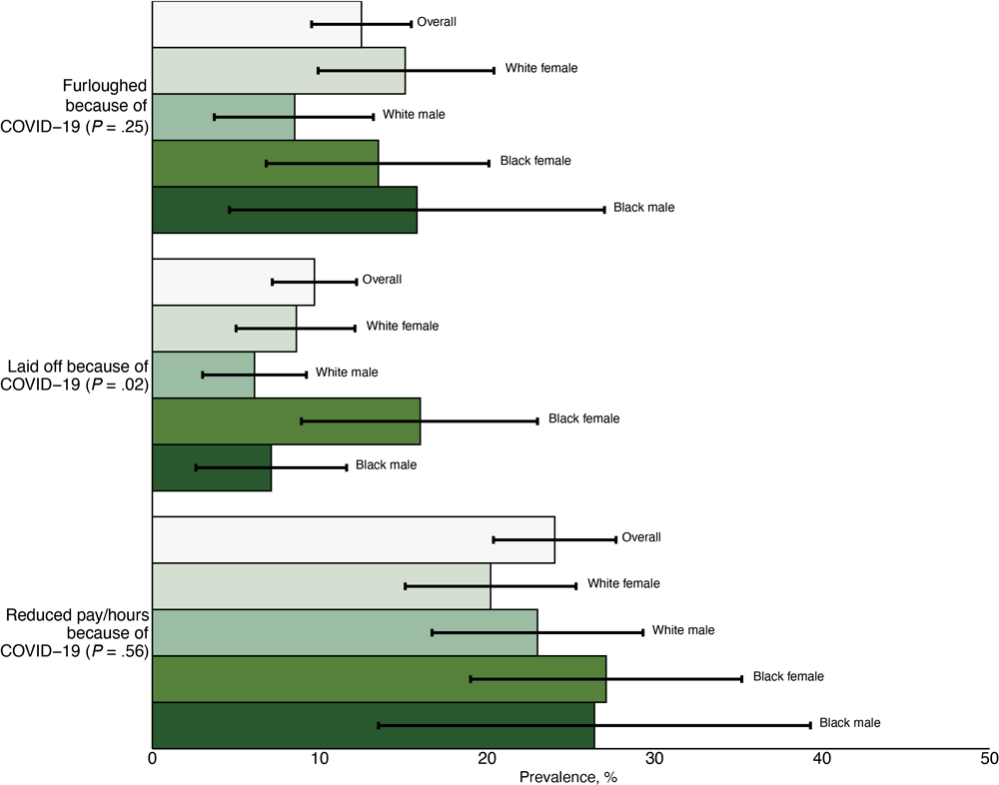
Estimated prevalence of 3 employment insecurity outcomes for St. Louis County residents and each sex and race subgroup. Prevalence is reported overall and for each race and sex subgroup. Group differences were assessed with a Rao-Scott χ^2^ *P*-value.

Although 9.7% (95% CI, 7.2%–12.2%) of respondents were estimated as laid off because of COVID–19, the estimate was higher for Black female respondents at 16% (95% CI, 8.9%–23.0%). Across other groups, 8.6% of White female respondents (95% CI, 5.0%–12.1%), 6.1% of White male respondents (95% CI, 3.0%–9.2%), and 7.1% of Black male respondents were estimated as laid off (95% CI, 2.6%–11.6%, *P* =.02). Both being furloughed and having reduced hours or pay did not differ across the 4 groups, with 12.5% (95% CI, 9.5%–15.5%, *P* = .25*)* and 24.0% (95% CI, 20.4%–27.7%, *P* = .56) of respondents estimated to have these employment changes, respectively. Weighted prevalence of employment loss among participants by race and sex is illustrated (Figure 1).

**Table:** 

Prevalence, 95% CI						
Outcome	Overall	White female	White male	Black female	Black male	*P*-value
Furloughed because of COVID-19	12.5 (9.5–15.5)	15.1 (9.9–20.4)	8.5 (3.7–13.2)	13.5 (6.8–20.1)	15.8 (4.6–27.0)	0.25
Laid off because of COVID-19	9.7 (7.2–12.2)	8.6 (5.0–12.1)	6.1 (3.0–9.2)	16.0 (8.9–23.0)	7.1 (2.6–11.6)	0.02
Reduced pay/hours because of COVID-19	24.0 (20.4–27.7)	20.2 (15.1–25.3)	23.0 (16.7–29.3)	27.1 (19.0–35.2)	26.4 (13.5–39.3)	0.56

**Figure 2:**
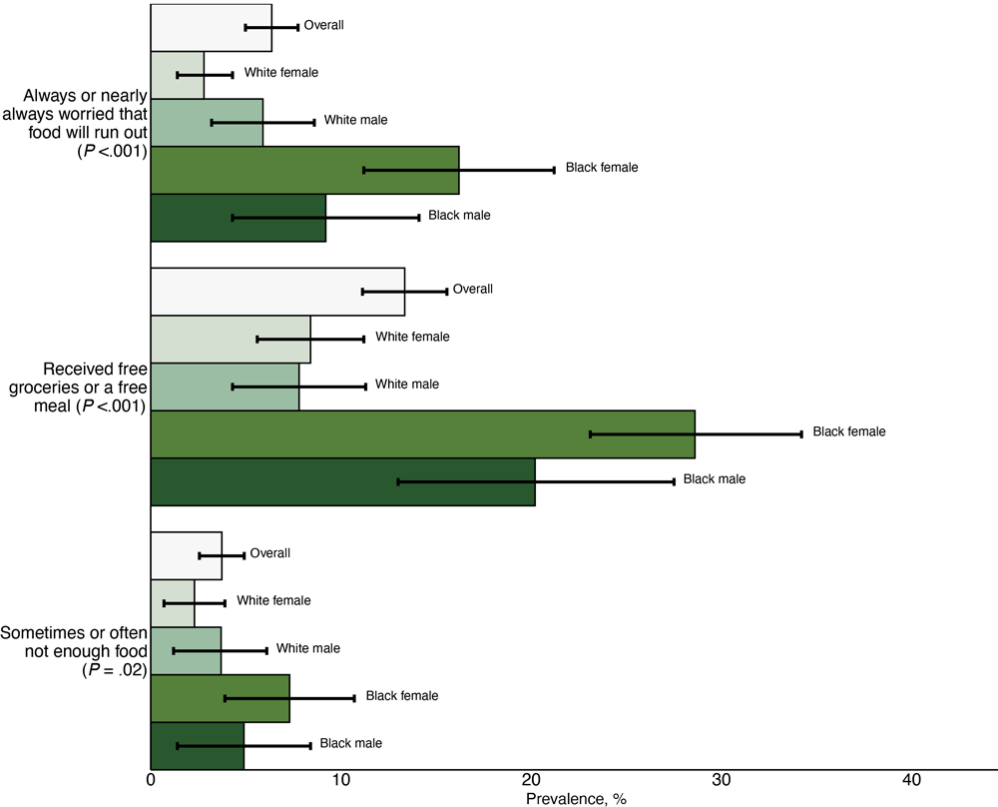
Estimated weighted prevalence for 3 food insecurity outcomes for St. Louis County residents and each sex and race subgroup. Prevalence is reported overall and for each race and sex subgroup. Group differences were assessed by using a Rao-Scott χ^2^ *P* value.

Relative to White males (80.7%; 95% CI, 75.6%–85.9%) and White females (85.3%; 95% CI, 81.8%–88.7%), and to Black males (85.3%; 95% CI, 79.6%–91.0%), Black females were estimated to have a lower prevalence (*P* = .02) of having enough food (73.8%; 95% CI, 68.3%–79.4%) (Figure 2). This pattern of differences for Black female residents was consistent on all food insecurity items. Black females were estimated to have had a higher estimated prevalence of having enough food but not type wanted (18.9%; 95% CI, 13.9%–23.8%), followed by White male residents (15.6%; 95% CI, 10.8%–20.4%) and White female residents (12.4%; 95% CI, 9.3%–15.6%). Black males were estimated to have the lowest prevalence of having enough food but not type wanted (9.8%; 95% CI, 5.2%–14.4%). Both Black females (28.6%; 95% CI, 23.1%–34.2%) and Black males (20.2%; 95% CI, 13.0%–27.5%) were estimated to have higher prevalences of receiving free meals or groceries compared with their counterparts (8.4% White females; 95% CI, 5.6%–11.2%) and (7.8% White males; 95% CI, 4.3%–11.3%, *P* < .001) (Figure 2).

**Table:** 

Prevalence, 95% CI						
Outcome	Overall	White female	White male	Black female	Black male	*P*-value
Always or nearly always worried that food will run out	6.4 (5.0–7.74)	2.8 (1.4–4.3)	5.9 (3.2–8.6)	16.2 (11.2–21.2)	9.2 (4.3–14.1)	<.001
Received free groceries or a free meal	13.3 (11.1–15.6)	8.4 (5.6–11.2)	7.8 (4.3–11.3)	28.6 (23.1–34.2)	20.2 (13.0–27.5)	<.001
Sometimes or often not enough food	3.7 (2.6–4.9)	2.3 (0.7–3.9)	3.7 (1.2–6.1)	7.3 (3.9–10.7)	4.9 (1.4–8.4)	0.02

The 4 groups also differed by frequency of worry that food will run out (*P* < .001). An estimated 74.4% (95% CI, 71.6%–77.2%) of St. Louis County residents never worried that food will run out. Frequencies were slightly above the average for White female residents (75.8%; 95% CI, 71.1%–80.5%) and male residents (79.6%; 95% CI, 74.6%–84.7%), and slightly below the average for Black male residents (72.6%; 95% CI, 64.9%–80.3%). Black female residents were estimated to have the lowest prevalence of never worrying that food will run out at 57.8% (95% CI, 51.6%–63.9%). Moreover, 4.3% (95% CI, 3.2%–5.4%) of St. Louis County residents were estimated to always worry that food will run out. Across each group, this rate was highest among Black female residents with 13.2% (95% CI, 8.4%–18.0%) always worrying.

### Sociodemographics as correlates of employment insecurity

For furlough, layoff, and reduced pay outcomes, there were no significant sociodemographic correlates. Regarding layoffs, although the overall model was not significant, Black female residents had higher odds of being laid off than White male residents. Specifically, Black female residents (OR = 2.61; 95% CI, 1.24–5.46, *P* = .05) had more than 2 times higher odds of being laid off, compared with White male residents (Table 2).

**Table 2:** Weighted Logistic Regression Models^a^ of COVID-19 Related Employment Loss Outcomes, St. Louis County, Missouri, August 12, 2020–October 27, 2020

Characteristics	COVID-19 furlough		COVID-19 lay-off		COVID-19 reduced pay or hours	
	OR (95% CI)	*P* ^ b^	OR (95% CI)	*P* ^ b^	OR (95% CI)	*P* ^ b^
**Sex and race subgroup**						
Black female	1.60 (0.72–3.53)	.35	2.61 (1.24–5.46)	.05	1.26 (0.73–2.16)	.51
Black male	1.79 (0.66–4.90)		0.97 (0.39–2.44)		1.25 (0.59–2.65)	
White female	1.98 (0.93–4.24)		1.46 (0.72–3.00)		0.85 (0.53–1.38)	
White male	1 [Reference]		1 [Reference]		1 [Reference]	
**Presence of children in household**						
Children in household	0.85 (0.44–1.64)	.63	1.05 (0.56–1.98)	.87	1.03 (0.66–1.60)	.90
No children in household	1 [Reference]		1 [Reference]		1 [Reference]	
**Age**	1.00 (0.98–1.03)	.89	1.00 (0.99–1.02)	.63	0.99 (0.98–1.01)	.24
**Education**						
≤High school diploma	1.51 (0.72–3.15)	.55	1.97 (0.95–4.08)	.18	0.78 (0.44–1.37)	.24
College 1–3 years	1.13 (0.55–2.33)		1.16 (0.61–2.18)		1.29 (0.81–2.05)	
College graduate	1 [Reference]		1 [Reference]		1 [Reference]	

a Values obtained through logistic regression with adjustment for all variables shown.

b Type 3 analysis of effects, *F* test, α = .05.

### Sociodemographics as correlates of food insecurity

Race and sex were significant predictors of receiving free meals or groceries. Compared with White male residents (*P* < .001), White female residents had similar odds of receiving free meals (OR = 1.00; 95% CI, 0.54–1.83), Black male residents had more than 2 times the odds (OR = 2.41; 95% CI, 1.15–5.07), and Black female residents had more than 4 times higher odds (OR = 4.13; 95% CI = 2.29–7.45). Additionally, the presence of children in the household was a significant predictor: residents with children present had 65% higher odds of receiving free meals or groceries (OR = 1.65; 95% CI, 1.05–2.58, *P* = .03) than those with no children in the household. Neither age (*P* = .52) nor education (*P* = .39) were found to be related to receipt of free meals or groceries. Employment was a predictor (*P* = .04), with those who were unemployed having a 77% higher odds of receiving free meals, compared with those who were employed (OR = 1.77; 95% CI, 1.05–2.98) (Table 3).

**Table 3:** Weighted Logistic Regression Models^a^ of COVID-19-Related Food Insecurity Outcomes, St. Louis County, Missouri, August 12, 2020–October 27, 2020

Characteristics	Received free groceries or a free meal during COVID-19		Quantity and quality of food eaten during COVID-19^b^			Worries about food running out before able to purchase more^c^		
	OR (95% CI)	*P* ^ d^	Enough food, but not type wanted, OR (95% CI)	Not enough food^e^, OR (95% CI)	*P* ^ d^	Sometimes, OR (95% CI)	Always or nearly always, OR (95% CI)	*P* d
**Race and sex subgroup**								
Black female	4.13 (2.29–7.45)	<.001	1.22 (0.73–2.06)	1.26 (0.45–3.48)	0.04	4.25 (2.28–7.94)	2.99 (1.52–5.87)	<.001
Black male	2.41 (1.15–5.07)		0.53 (0.25–1.10)	0.75 (0.24–2.39)		1.44 (0.69–3.00)	1.19 (0.52–2.75)	
White female	1.00 (0.54–1.83)		0.73 (0.46–1.15)	0.47 (0.15–1.54)		1.93 (1.04–3.60)	0.43 (0.20–0.93)	
White male	1 [Reference]		1 [Reference]	1 [Reference]		1 [Reference]	1 [Reference]	
**Presence of children in household**								
Children in household	1.65 (1.05–2.58)	0.03	1.14 (0.74–1.76)	1.82 (0.71–4.72)	0.42	1.72 (1.06–2.80)	1.68 (0.91–3.09)	0.04
No children in household	1 [Reference]		1 [Reference]	1 [Reference]		1 [Reference]	1 [Reference]	
**Age**	1.00 (0.98–1.01)	0.52	1.00 (0.99–1.01)	1.01 (0.98–1.04)	0.93	1.00 (0.99–1.02)	0.99 (0.98–1.01)	0.67
**Education**								
High school diploma or less	1.46 (0.84–2.55)	0.39	1.26 (0.72–2.21)	3.46 (1.45–8.23)	0.01	1.59 (0.89–2.86)	2.03 (0.99–4.15)	0.22
College 1–3 years	1.10 (0.69–1.76)		1.36 (0.85–2.15)	3.78 (1.63–8.78)		1.16 (0.67–2.01)	1.67 (0.87–3.20)	
College graduate	1 [Reference]		1 [Reference]	1 [Reference]		1 [Reference]	1 [Reference]	
**Employment**								
Retired	0.86 (0.53–1.40)	0.04	0.65 (0.40–1.07)	0.85 (0.32–2.25)	0.02	0.52 (0.26–1.04)	1.05 (0.52–2.12)	0.01
Unemployed	1.77 (1.05–2.98)		1.07 (0.64–1.82)	4.02 (1.55–10.39)		1.48 (0.84–2.62)	2.37 (1.27–4.41)	
Employed for wages	1 [Reference]		1 [Reference]	1 [Reference]		1 [Reference]	1 [Reference]	

a Values obtained through simple (received free groceries or a free meal during COVID-19) or multinomial (quantity and quality of food eaten during COVID-19, worry about food running out before ability to purchase more) logistic regression with adjustment for all variables shown.

b Outcome reference response: Enough food.

c Outcome reference response: Seldom or never.

d Type 3 analysis of effects, *F* test, *α* = .05.

e Sometimes or often not enough food.

Relative to White male residents (*P* = .04), White females had 27% lower odds (OR = 0.73; 95% CI, 0.46–1.15) and Black males had 47% (OR = 0.53; 95% CI, 0.25–1.10) lower odds of having enough food, but not type wanted. Black females had 22% times higher odds (OR = 1.22; 95% CI, 0.73–2.06). Similarly, White females (OR = 0.47; 95% CI, 0.15–1.54) and Black males (OR = 0.75; 95% CI, 0.24–2.39) had lower odds of sometimes or often not having enough food compared with White males; Black female residents had 26% higher odds of sometimes or often not having enough food compared with White males (OR = 1.26;, 95% CI, 0.45–3.48). Furthermore, compared with those with a 4-year college degree (*P* =.01), residents with a high school education or less had 26% higher odds of having enough food but not type wanted (OR = 1.26; 95% CI, 0.72–2.21) and more than 3 times higher odds of not having enough food sometimes or often (OR = 3.46; 95% CI, 1.45–8.23). Residents with some college had 36% (OR = 1.36; 95% CI, 0.85–2.15) higher odds of having enough food but not type wanted, and more than 3 times higher odds of not having enough food sometimes or often (OR = 3.78; 95% CI, 1.63–8.78). Additionally, compared with employed residents (*P* = .002), those who were unemployed had 4 times higher odds of not having enough food sometimes or often (OR = 4.02; 95% CI, 1.55–10.39). 

Compared with White male residents (*P* < .001), White females had nearly 2 times higher odds of sometimes worrying about food (OR = 1.93; 95% CI, 1.04–3.60), although Black males had 44% higher odds (OR = 1.44; 95% CI, 0.69–3.00) and Black females had more than 4 times the odds (OR = 4.25; 95% CI, 2.28–7.94). Regarding always or nearly always worrying about food, White females had 57% lower odds of worry, compared with White males (OR = 0.43; 95% CI, 0.20–0.93). Black males had 19% higher odds (OR = 1.19; 95% CI, 0.52–2.75), and Black females had nearly 3 times higher odds of always or nearly always worrying about food compared with White males (OR = 2.99; 95% CI, 1.52–5.87). Compared with households without children (*P* = .04), those with children had 72% higher odds of sometimes worrying about food (OR = 1.72; 95% CI, 1.06–2.80). Although neither age nor education were found to be predictors of food worry (*P* = .67 and *P* = .22, respectively), employment status was significant (*P* = .01), such that those unemployed had 2 times higher odds of always worrying about food than those employed (OR = 2.37; 95% CI, 1.27–4.41) (Table 3).

## Discussion

The aim of this study was to investigate the relationship between sociodemographic characteristics and 2 important social determinants of health, employment loss and food insecurity, during the COVID-19 pandemic among Black and White adults living in Saint Louis County, Missouri. We separately analyzed both employment loss and food insecurity and found that Black adult residents were disproportionately affected, compared with White adults. Additionally, we observed that Black females experienced the greatest burden of economic hardships.

These results corroborate findings from an emerging body of literature demonstrating the excessive burden of COVID-19 among Black Americans generally (18), and among Black women more specifically (19–21). We emphasize, however, that these are not new challenges for Black women, but long-standing systemic social and economic injustices against this group on the basis of their interlocking identities of being both Black and female (14,22). Because of their intersectional oppressions, Black women experience racism and sexism that make them more likely to be segregated into low-wage occupations that offer inadequate benefits, workplace inflexibility, and job insecurity (23,24). In the context of COVID-19, these sex and race inequities have placed a disproportionate number of Black women on the frontlines, working in jobs that cannot be done from home, which places them at higher risk of potential COVID-19 infections, hospitalizations, and deaths (21).

In our study, Black women were more likely to be laid off compared with White men and most likely to always worry about food more than the other groups. These findings suggest that COVID-19 created more social risks and distress for Black females and highlights a need for additional support for this population. Further, Black females typically have multiple primary caregiving responsibilities, and they provide support for both their nuclear and extended family systems, as well as friends and fictive kin (people not biologically or legally related yet who are considered to be “family”) (25).

Compared with White women, Black women are more likely to provide this care in isolation without the help of others and to experience more financial hardships as a result of their caregiving (23). Without adequate systems and policies to support Black women, it is conceivable that entire family and friend networks supported by Black women are placed at increased risks of food insecurity and other adverse social conditions.

We observed that the estimated overall prevalence of food insecurity in St. Louis County residents increased since the beginning of the pandemic until the end of our study. Moreover, in 2019 (pre-pandemic), 10.1% of all St. Louis County residents were food insecure, and our findings show slightly higher rates, for example, 13.3% of residents receiving free groceries or meals (26). Among those who were food insecure, Black respondents living with children and those who were unemployed were more likely to receive assistance in the form of free groceries or meals, supporting prior study findings (27). Given the higher prevalence of pre-existing food insecurity among these groups, it is possible that they were already familiar with accessing and using community resources from needs before the pandemic. Formerly established social networks and community ties might have provided them with the advantage to know more readily where and how to access needed resources during the pandemic (28,29).

Our findings are consistent with other evidence documenting the protective benefits of a college-level education to buffer against the social and health harms of COVID-19 (27). Respondents in our sample with a high school education or less were more vulnerable to being laid off from their jobs and being food insecure since COVID-19.

Our study has limitations. The cross-sectional design limits causal conclusions. The study also does not account for whether people had pre-existing food insecurity or employment hardships compared with new hardships since the pandemic. Groups having new hardships since the pandemic or existing hardships before the pandemic may be different in important ways that were not explored in this study. Another limitation of this study is low response rates. Although weighting techniques were applied to reduce bias and obtain a more representative sample, estimated proportions of residents in St. Louis County affected by food insecurity or employment loss may still be underestimated or overestimated. Additionally, racial and ethnic groups other than Black or White, and people who did not identify as male or female, were not included in our sample, limiting our understanding of how COVID-19 affected employment loss and food insecurity for these groups. Despite these limitations, our analysis had strengths. Our study decreased digital divide challenges in reaching participants by requiring only a cellular telephone or landline to be eligible. Given the large sample size and the complex sampling design, our findings are likely to be generalizable to adults living in similar types of counties in the US. Furthermore, the study is timely, and was administered during the pandemic to assess COVID-19–related concerns occurring in “real-time.” The findings suggest additional research is needed to identify factors that contribute to elevated social harms in the context of a pandemic. For instance, given the disproportionate rates of chronic conditions like heart disease and diabetes among Black women compared with White women (21), it is possible that if unable to work from home, these women may have had to decide between their financial wellness or physical wellness, and chose, or were forced to choose, to exit their employment.

Moreover, this study sheds light on group differences by race and sex, providing further insight beyond studies examining only gender or only racial disparities in employment loss and food insecurity. Identifying which segments of the population are more likely to experience increased social harms is critical to prevent a subsequent increase in chronic disease incidence, morbidity, and mortality (30). In summary, this study provides important and relevant contributions and insights into the uneven social harms associated with the COVID-19 pandemic on different population segments. Results can be used to guide programs, interventions, and policies to mitigate the disproportionate impact of COVID-19 and its related social harms on Black women.
